# Use of selective serotonin-reuptake inhibitors in the first trimester and risk of cardiovascular**-**related malformations: a meta-analysis of cohort studies

**DOI:** 10.1038/srep43085

**Published:** 2017-02-21

**Authors:** Tie-Ning Zhang, Shan-Yan Gao, Zi-Qi Shen, Da Li, Cai-Xia Liu, Hai-Chen Lv, Yuan Zhang, Ting-Ting Gong, Xin Xu, Chao Ji, Qi-Jun Wu

**Affiliations:** 1 Department of Pediatrics, Shengjing Hospital of China Medical University, Shenyang, 110004, China; 2 Department of Clinical Epidemiology, Shengjing Hospital of China Medical University, Shenyang, 110004, China; 3 Department of Obstetrics and Gynecology, Shengjing Hospital of China Medical University, Shenyang, 110004, China; 4 Department of Cardiology, First Affiliated Hospital of Dalian Medical University, Dalian, 116011, China; 5 Department of Emergency, Shengjing Hospital of China Medical University, Shenyang, 110004, China

## Abstract

The relationship between selective serotonin-reuptake inhibitors (SSRIs) use during first trimester and cardiovascular-related malformations of infants is still uncertain. Therefore, we conducted this systematic review and meta-analysis to assess the aforementioned association. A systematic literature review identified studies for cohort studies about SSRIs use and cardiovascular-related malformations in PubMed and Web of Science. We summarized relative risk (RRs) and 95% confidence intervals (CIs) of cardiovascular-related malformations using random-effects model, and heterogeneity and publication-bias analyses were conducted. Eighteen studies met the inclusion criteria. Pregnant women who were exposed to SSRIs at any point during the first trimester had a statistically significant increased risk of infant cardiovascular-related malformations (RR = 1.26, 95%CI = 1.13–1.39), with moderate heterogeneity (I^2^ = 53.6). The corresponding RR of atrial septal defects (ASD), ventricular septal defects (VSD), ASD and/or VSD was 2.06 (95%CI = 1.40–3.03, I^2^ = 57.8), 1.15 (95%CI = 0.97–1.36; I^2^ = 30.3), and 1.27 (95%CI = 1.14–1.42; I^2^ = 40.0), respectively. No evidence of publication bias and significant heterogeneity between subgroups was detected by meta-regression analyses. In conclusion, SSRIs use of pregnant women during first trimester is associated with an increased risk of cardiovascular-related malformations of infants including septal defects. The safety of SSRIs use during first trimester should be discussed to pregnant women with depression.

Depression in pregnancy is common^1^. Approximately 10% of pregnant women experience depression^2^ and up to 15% of pregnant women experience depression symptoms^3^,^4^. Since untreated depression may have potential risk to offspring, such as restricted fetal growth^5^,^6^, low birth weight^7^, and lower child body mass index (BMI)^8^, it is essential to manage antenatal depression^9^. After the introduction of SSRIs into the market, SSRIs have become the most commonly prescribed pharmacological treatment for depression during pregnancy^10^,^11^, which was considered relatively safe to take during pregnancy prior to 2005^12^. The study carried out by Einarson *et al*. as well as a meta-analysis and two database failed to find SSRIs was associated with a risk of major malformations^12^,^13^,^14^,^15^. However, the studies^16^,^17^ published after 2005 reported that SSRIs might increase the incidence of cardiovascular malformations in infants. Since approximately 75% of pregnant women who experienced depression were treated with SSRIs in the first trimester of pregnancy^18^, the safety of SSRIs use has attracted more attention and has become a clinical issue as a result.

The association between SSRIs use during pregnancy and birth defects risk has still been controversial. There have been many studies conducted to determine the association between SSRIs use and cardiovascular malformations. Some studies reported maternal SSRIs use was related to an increased risk of congenital cardiac malformations^19^,^20^,^21^,^22^, while other studies suggested there was no association^12^,^23^,^24^. Three meta-analyses^25^,^26^,^27^ demonstrated that SSRIs use during pregnancy did not increase the risk of major or minor cardiovascular malformations. By contrast, other two meta-analyses^28^,^29^ reported that SSRIs were associated with an increased risk of cardiovascular malformations. However, several limitations were observed in these five meta-analyses: (1) case-control studies were included which might generate more bias (selection and information bias), (2) the exposure period of SSRIs of pregnant women was not consistent. For example, the meta-analysis carried out by Grigoriadis *et al*.^29^ included studies with different exposure periods of SSRIs (e.g., first, second, third, and term trimester), (3) the inclusion and exclusion criteria were not unify. For example, the meta-analysis conducted by Wang *et al*.^27^ only included four studies which were relatively small part of these studies should be included and (4) whether findings were robust in subgroup analyses stratified by adjustment for potential confounders were limited.

Additionally, cohort studies^30^,^31^,^32^,^33^,^34^,^35^,^36^ with larger number of populations have been conducted to explore the aforementioned association in Europe and North America recently. For example, in 2015, a prospective cohort study conducted by Berard *et al*.^31^ reported that there was no association between maternal use of SSRIs during pregnancy and cardiac malformations whose risk ratio of cardiac malformations was 1.10 (95%CI = 0.82–1.48). However, Furu *et al*.^30^ reported that, in a prospective cohort study which contained 2,303,647 singleton live births in Nordic countries including Denmark, Finland, Iceland, Norway, and Sweden, cardiac defects was associated with SSRIs use during pregnancy whose odds ratio of any cardiac defect was 1.15 (95%CI = 1.05–1.26). In order to better understand the relationship between SSRIs use in pregnant women during the first trimester and cardiovascular-related malformations of infants, we performed a systematic review and meta-analysis using data from cohort studies.

## Results

### Literature search

**Figure 1:**
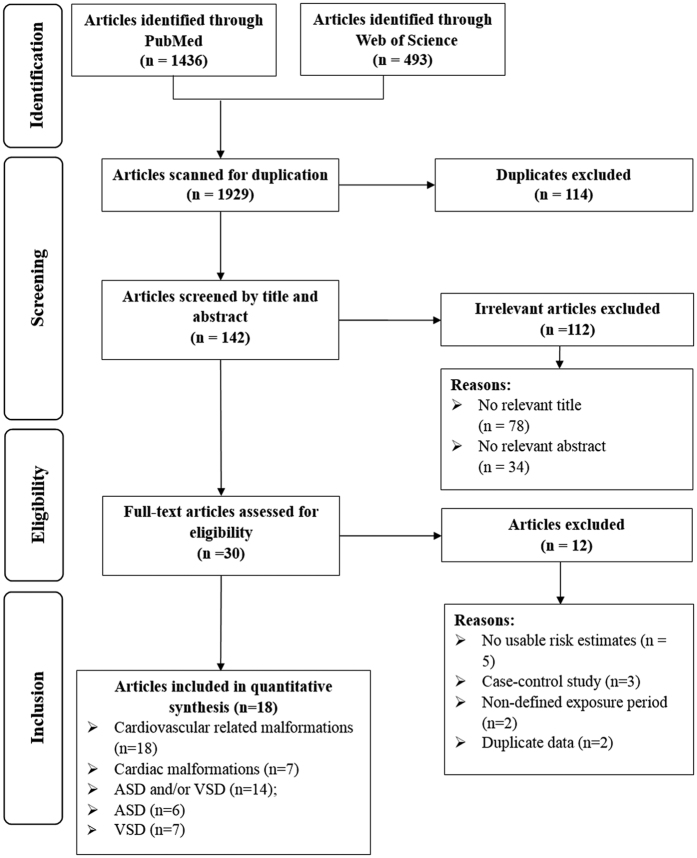
Flow-chart of study selection.

We identified 1436 studies from PubMed and 493 studies from Web of science via the search strategy. Of all studies, 1929 studies were excluded after we screened the titles and abstracts for the general criteria. Only 18 studies were eligible for inclusion into the final meta-analysis^19^,^20^,^21^,^22^,^30^,^31^,^32^,^33^,^34^,^35^,^36^,^37^,^38^,^39^,^40^,^41^,^42^,^43^ after the full text was reviewed for the 30 potential studies (Fig. 1).

### Study characteristics

Characteristics of these 18 studies are shown in Supplementary Table S1. These studies were published between 1998 and 2015. Of all these included studies, eleven studies were conducted in Europe^19^,^30^,^32^,^34^,^35^,^36^,^37^,^38^,^39^,^40^,^42^, five studies were conducted in Northern America^22^,^31^,^33^,^41^,^43^, and one study each were conducted in Australia^21^ and Israel^20^. Sample sizes of these studies ranged from 534 to 2,303,647, and the number of cardiovascular-related malformations varied from 6 to 27,309.

### Quality of included studies

Supplementary Table S2 presents the study-specific quality according to Newcastle-Ottawa quality scale^44^. In the ‘control for important factor or additional factor’ category, nine studies^20^,^21^,^22^,^31^,^33^,^34^,^38^,^41^,^43^ were not assigned two scores because they adjusted, less than two, important confounders in their primary analyses. In the ‘follow-up long enough for outcomes to occur’ and ‘adequacy of follow-up of cohorts’ categories, six equivalent studies^22^,^32^,^37^,^38^,^42^,^43^ were not assigned a score because they did not refer to follow-up in their studies. The maximum score is nine and the minimum score is five.

### Cardiovascular-related malformations

**Figure 2:**
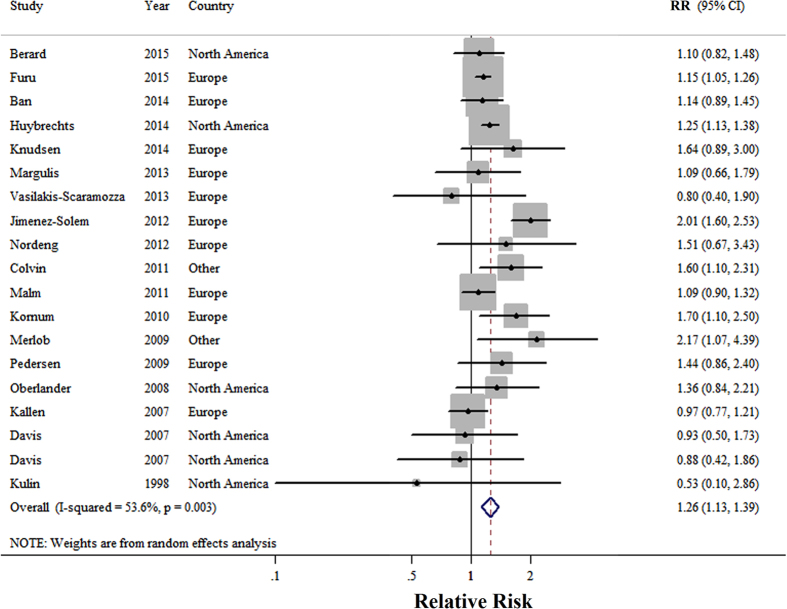
Forest plots of the relationship between SSRIs use and risk of cardiovascular related malformations. Squares indicate study-specific risk estimates (size of the square reflects the study-specific statistical weight); horizontal lines indicate 95% CIs; diamond indicates the summary relative risk with its 95% CI. RR: relative risk.

**Figure 3:**
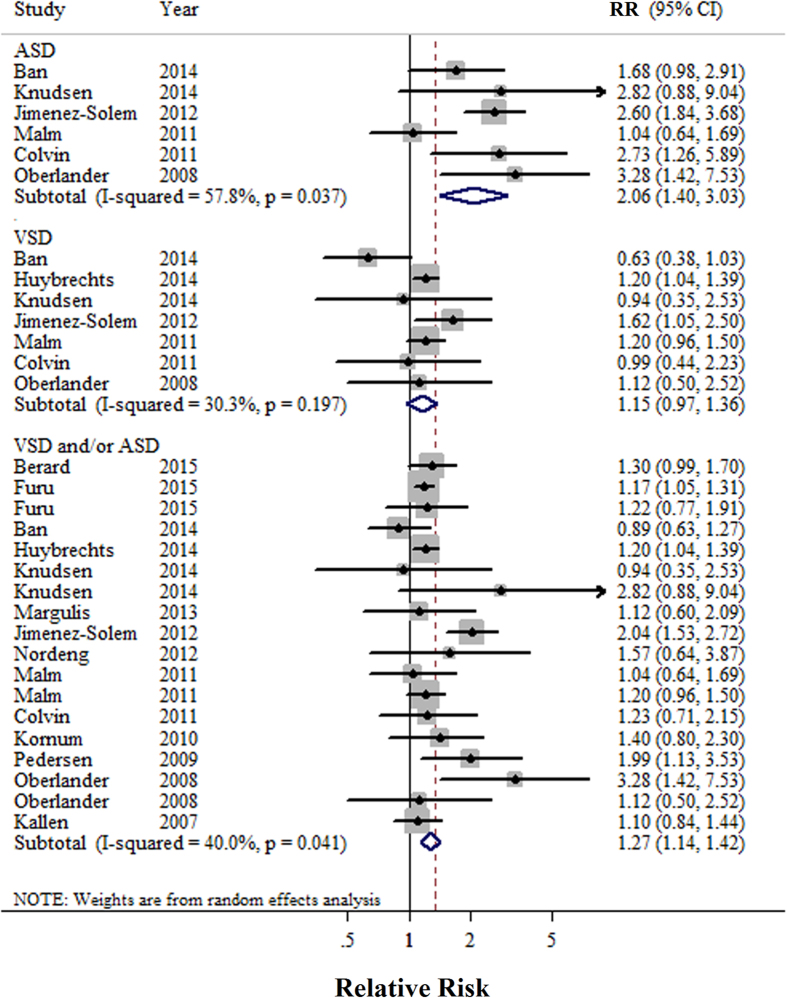
Forest plots of the relationship between SSRIs use and risk of septal defects. Squares indicate study-specific risk estimates (size of the square reflects the study-specific statistical weight); horizontal lines indicate 95% CIs; diamond indicates the summary relative risk with its 95% CI. ASD: atrial septal defect; RR: relative risk; VSD: ventricular septal defect.

A total of 18 studies^19^,^20^,^21^,^22^,^30^,^31^,^32^,^33^,^34^,^35^,^36^,^37^,^38^,^39^,^40^,^41^,^42^,^43^ reported the association between SSRIs use of pregnant women during first trimester and cardiovascular-related malformations of infants. Pregnant women who were exposed to SSRIs at any point during the first trimester had a statistically significant increased risk of infant cardiovascular-related malformations (RR = 1.26, 95%CI = 1.13–1.39), with moderate heterogeneity (P = 0.003, I^2^ = 53.6) (Fig. 2). There was no indication of a publication bias according to Begg’s test (P-bias = 0.889) or Egger’s test (P-bias = 0.601), and there was no asymmetry in funnel plots when inspected visually. Additionally, analyses of the seven studies^30^,^31^,^33^,^36^,^40^,^42^,^43^ that reported cardiac malformations per our criteria showed that there was a statistically significant increased risk of cardiac malformations in infants born to mothers who used SSRIs in the trimester (RR = 1.17, 95%CI = 1.06–1.28; P = 0.19, I^2^ = 31.0). Furthermore, analyses of studies reported the cardiac malformations of interest were ASD^21^,^22^,^30^,^32^,^34^,^37^,^39^, VSD^21^,^22^,^32^,^33^,^34^,^37^,^39^ and ASD and/ or VSD^19^,^21^,^22^,^30^,^31^,^32^,^33^,^34^,^36^,^37^,^38^,^39^,^40^,^42^ with corresponding RR scores of 2.06 (95%CI = 1.40–3.03), 1.15 (95%CI = 0.97–1.36), and1.27 (95%CI = 1.14–1.42) respectively (Fig. 3).

### Subgroup and sensitivity analysis

We performed subgroup analyses in terms of geographic location and potential for confounded adjustment (Table 1). When stratified by geographic location, all strata showed significant results. Additionally, although the directions of the results of subgroup analyses stratified by potential confounders were consistent with the main findings, not all of them showed statistically significance.

In a sensitivity analysis, we evaluated the effect of removing a single study from the total, for each of the 18 studies, in order determine its effect on the summarized estimate for heterogeneity and to assess whether one study had a significant influence on the meta-analytic RR. The 18 study-specific RRs of cardiovascular-related malformation ranged from a low of 1.19 (95%CI = 1.10–1.28; P = 0.25, I^2^ = 17.3%) after omission of the study by Jimenez-Solem *et al*.^39^ to a high of 1.29 (95%CI = 1.16–1.44; P = 0.005, I^2^ = 53.7) after omission of the study by Kallen *et al*.^42^.

## Discussion

In this meta-analysis of 18 cohort studies, we found that SSRIs use during the first trimester increase the risk for cardiovascular and septal defects malformations by 26% and 27%, respectively. The same positive findings were also observed in most of the subgroup analyses. Considering the high prescription rate of SSRIs in pregnant women with depression, the safety of SSRI should be discussed with women in the first trimester.

The biological mechanisms of SSRIs use and cardiovascular-related malformations are still unclear thus far. SSRIs could cross the placenta and may, therefore, increase the incidence of fetal heart defect and alter placental and fetal heart serotonin signaling^45^. Experimental study indicates that myocardial staining in mice embryos exposed to serotonin restricts the development endocardial cushion forming regions and was became almost completely blocked with uptake inhibitors. This process appeared to be mediated by serotonin transporters^46^. The blockage of serotonin uptake by paroxetine could decrease the number of 5-bromo-deoxyuridine immunoreactive and MF20-im cells, and this data indicates that serotonin and serotonin transporters has a significant role in heart development^47^. Another study, conducted by Buskohl *et al*.^48^, reported elevated serotonin in avian induced atrioventricular valvuloseptal defect *in vivo*. Severe heart defects may be induced via a transforming growth factor-beta/serotonin signaling pathway. Since there is a relationship between signaling networks and cell/tissue level, and little information about other signal pathways, more research into the biological mechanism between SSRIs and cardiovascular-related defects should be pursued. Besides, a study conducted by Lage *et al*.^49^ showed that genetic and environmental risk factors modulate critical biological systems during heart development, especially influencing protein networks driving the development of specific anatomical structures. Therefore, further research should pay more attention to the influence of environmental and epigenetic factors between SSRIs and cardiovascular-related defects.

Although all results of subgroup analyses stratified by geographic locations showed significance, the point estimates were slightly different. This might be attributed to the fact that different populations may have different exposure rates of SSRIs. Berard *et al*.^31^ reported the exposure rate of SSRIs in pregnant women was 12.59% based on 18,493 participants between 1998 and 2010 from Quebec Pregnancy Cohort in Canada; whereas Margulis *et al*.^36^ reported the exposure rate was 2.40% on the basis of 149,464 participants between 1996 and 2010 from the Clinical Practice Research Datalink’s Mother Baby Link in UK. By comparison, the aforementioned rate was 3% and 0.35% for pregnant women from Australia^21^ and Israel^20^, respectively. Additionally, several studies reported SSRIs on the basis of several specific antidepressants, which might result in the different results of geographic locations. For example, Furu *et al*.^30^ noted that in the studies conducted by Huybrechs *et al*.^33^, seven kinds of SSRIs were contained in their study including fluoxetine, citalopram, paroxetine, sertraline, fluvoxamine, escitalopram and venlafaxine, whereas there were only three kinds of SSRIs including paroxetine, sertraline and fluoxetine.

The strengths of this meta-analysis include: the large sample size of 7,280,932 participants, and excluding cases that may bias the results. This sample size was chosen to provide sufficient statistical power to detect the association between SSRIs use and cardiovascular-related malformations In addition, because we only included cohort studies in the present study, recall and selection bias is not likely to affect the results. Moreover, compared with previous meta-analyses, numerous subgroup and sensitivity analyses were carried out to explore heterogeneity of the data.

Despite the clear strengths of this study, some limitations of our study should be acknowledged. First of all, in almost all studies, the data only consisted of live births and therefore lacked information about pregnancies that did not end with a live birth, such termination of pregnancy, stillbirth, or miscarriages. If pregnant women exposed to SSRIs had a higher incidence rate of abortions as a result of the severe heart malformations and defects, it could mask the teratogenic effect of SSRIs and introduce an unintentional selection and detection bias. Pregnant women exposed to SSRIs were reported to have an increased rate of taking ultrasound examinations compared with the women not exposed to SSRIs^50^. More frequent ultrasound examinations could also increase the risk of congenital heart defects detection and the detection of malformations could also lead to pregnancy termination.

Secondly, several studies failed to control for potential confounders, which might introduce bias in an unpredictable direction. In fact, there are some known or suspected risk factors for cardiac defects such as age of delivery, state of residence, age, race, and parity, etc^33^. However, these potential confounders were not consistent in each study. Some studies did not adjust for any confounders while others adjusted for non-consistent confounders. For example, Colvin *et al*.^21^ did not note the adjustment for any potential confounders in their results while Berard *et al*.^31^ and Ban *et al*.^32^ adjusted for six and nine kinds of potential confounders, respectively. In addition, the study^30^ conducted by Furu *et al*. use the sibling analysis adjusting more potential confounders (e.g. family related factors), but these attenuated results were generated from only 2,288 participants which might be attributed to limited statistical power when comparing to primary cohort (n = 2,303,647). Besides, some specific confounding factors such as maternal BMI or obesity, which seems to itself increase the risk of congenital heart defects including the septal ones^51^,^52^,^53^. Any further studies should fully adjust these potential confounders or report analyses stratified by these risk factors to better be able to rule out residual confounding.

Thirdly, because the majority of included studies (15/18)^19^,^20^,^21^,^22^,^30^,^31^,^32^,^33^,^34^,^35^,^36^,^38^,^40^,^42^,^43^ were based on register data, we could not get information on diagnostic tests for all cardiovascular-related malformations. Although echocardiography has been the most useful diagnostic test to confirm the presence of congenital heart defects^54^ in utero, it greatly dependent on the clinical skills and knowledge of operators. Therefore, the comparison of detection rates varies according to an operators’ ability. Additionally, although most articles referenced the International Classification of Diseases (ICD), different revisions were used, for example Ninth Revision (ICD-9) or Tenth Revision (ICD-10), to identify the malformations. We failed to get a uniform consensus in cardiovascular-related malformations containing all of the conditions for birth defects of the circulatory system. For example, 4 studies^21^,^22^,^35^,^38^ used “cardiovascular anomalies” to describe any circulatory defect, whereas 7 studies^30^,^31^,^33^,^36^,^40^,^42^,^43^ used “cardiac defect” or “cardiac malformation.” Further studies should establish consistent definitions of cardiovascular-related diseases by explicitly defining every kind of disease included in overall outcomes to reduce bias among different studies.

Finally, the estimates based on blank control group were included in our meta-analysis when the studies presented different measures of association. For example, the study conducted by Huybrechts *et al*.^33^ presented three kinds of estimates with increasing levels of confounding adjustments, but the adjusted estimates were based on pregnant women with depression. Therefore, we chose crude estimates instead of adjusted estimates.

## Conclusion

Our meta-analysis suggests that SSRIs use in pregnant women during first trimester is associated with an increased risk of cardiovascular-related malformations of infants including septal defects. Additional studies are needed to provide more detailed results, including research into every possible SSRI that is used by pregnant with results stratified by the different kinds of cardiovascular-related congenital defect after better adjustment for any potential confounders.

## Methods

### Literature search

We followed the guideline of the Preferred Reporting Items for Systematic reviews and Meta-Analyses^55^ to perform and report this meta-analysis. We conducted computerized literature searches of the databases including PubMed and Web of Science and reviewed the data from the database index date through December 31, 2015. The following search key words and Medical Subject Heading (MeSH) terms were used: (serotonin reuptake inhibitors OR SSRI OR fluoxetine OR paroxetine OR citalopram OR escitalopram OR sertraline OR fluvoxamine) AND (malformations OR birth outcome OR obstetrical outcome OR congenital abnormalities). Additionally, the references cited in the retrieved articles were scrutinized by manual search.

### Study selection

The studies that were included were considered if they met the following criteria: (1) used cohort study design, (2) defined the exposure period of SSRI as the first trimester of pregnancy, (3) defined the non-exposure group as normal pregnant women who did not use any antidepressant drug during first trimester, (4) reported any cardiovascular-related malformations at birth, (5) reported any usable risk estimates (e.g., odds ratio, risk ratio or relative risk with 95% CIs or necessary data for calculation) of the association between SSRI use and cardiovascular malformations.

The studies were excluded if they: (1) were review articles, systemic review and meta-analysis, commentaries, editorials or meeting abstracts, (2) used non-cohort study designs (e.g., case-control study, descriptive study, randomized controlled trial, etc.), (3) did not define the exposure period of SSRI as the first trimester, (4) included pregnant women who were exposed to two kinds of anti-depressants or more at the same time, (5) used pregnant women who took any antidepressant drug as reference group, (6) were not human studies or published in English.

When duplicate articles from the same study were identified, we included the most recent report that contained the largest number of the cohorts or cases that matched our interest. The selection and exclusion were carried out by 2 independent researchers (T-NZ and Z-QS). Disagreements were discussed and agreed-upon prior to selection.

### Data extraction

Data was independently extracted according to a standardized format by 2 researchers (T-NZ and Z-QS) for each eligible study. Disagreements were resolved by a third researcher (Q-JW) through discussion. From each study, we extracted the information as follows: first author, year of publication, geographic location, sample size (cases and cohort size), study period, outcome with their risk estimates and 95%CIs. Since there were only a limited number of studies with the specific outcomes of interest (e.g., atrioventricular septal defects, transposition of great arteries, situs anomalies and looping defect, etc), we summarized and presented the outcomes that were generally cardiovascular anomalies, cardiac malformations, and septal defects (ASD and/or VSD). We also extracted the adjusted confounders information of each study. If there were multiple estimates of the association, we extracted the estimate that was adjusted for the largest number of potential confounders. If there was no adjusted estimate in the study, we used a crude estimate.

### Quality evaluation

Two independent researchers (T-NZ and S-YG) conducted the quality assessment of these included studies according to the Newcastle-Ottawa Scale (NOS) for cohort studies^56^,^57^,^58^,^59^. All 8 items in the scale were applicable to our study question. The items can be divided into 3 domains (i.e., selection, comparability, and exposure/outcome). We used these NOS parameters to evaluate the studies instead of scoring them and categorizing them into high- or low quality on the basis of the scores.

### Statistical analysis

The studies^19^,^20^,^21^,^22^,^30^,^31^,^32^,^33^,^34^,^35^,^36^,^37^,^38^,^39^,^40^,^41^,^42^,^43^, reported the outcomes of specific heart anomalies e.g. any cardiac defect, cardiac malformations, congenital heart defects, cardiovascular anomalies, all major cardiovascular anomalies, bulbus cordis anomalies and anomalies of cardiac septal closure, and other congenital anomolies of heart. We extracted this data in order to calculate the summarized overall RR. The studies^19^,^21^,^22^,^30^,^31^,^32^,^33^,^34^,^36^,^37^,^38^,^39^,^40^,^42^, reported the outcomes of ASD (including ostium secundum type atrial septal defect), VSD, septal defect, atrioventricular septal defect, and ASD and/or VSD. We extracted this data in order to calculate the summarized RR of ASD and/or VSD events. For the study^31^, that separately reported the risk estimates of SSRIs but did not combine them, we used the effective count method proposed by Hamling *et al*.^60^ to recalculate the total risk estimate^61^,^62^,^63^,^64^. We reported all results in terms of the RR for simplicity since absolute risk of cardiovascular malformations are low. If there was no estimate specified in a study, we calculated it by using the original data from the study^22^,^43^. We calculated summarized RRs and 95%CIs by using the random effects model described by DerSimonian and Laird^65^. The I^2^ statistic was used to evaluate the heterogeneity of RRs across studies and we considered the values 50% or less, 51–75% and 76% or more as low-, moderate-, and high-heterogeneity, respectively^66^,^67^,^68^. Subgroup analysis was carried out on the basis of the geographic location (Europe, Northern America, and other regions). Additionally, we also stratified the meta-analysis by potential confounders including age, socioeconomic status, pregnancy body mass index, pregnancy complications and parity. Heterogeneity between subgroups was evaluated by meta-regression analysis. We also performed sensitivity analyses by excluding one study at a time to explore whether results were strongly influenced by a specific study. Finally, publication bias was evaluated through Egger’s linear regression^69^, Begg’s rank-correlation methods^70^, and funnel plots. We assumed that there was a significant statistical publication bias if P is less than 0.05 for Egger’s or Begg’s test. All statistical analyses were performed with Stata 12.1 (StataCorp).

## Additional Information

**How to cite this article:** Zhang, T.-N. *et al*. Use of selective serotonin-reuptake inhibitors in the first trimester and risk of cardiovascular-related malformations: a meta-analysis of cohort studies. *Sci. Rep.* **7**, 43085; doi: 10.1038/srep43085 (2017).

**Publisher's note:** Springer Nature remains neutral with regard to jurisdictional claims in published maps and institutional affiliations.

## Supplementary Material

Supplementary Tables

## Figures and Tables

**Table 1:** Summary risk estimates of the association between selective serotonin reuptake inhibitor use and cardiovascular-related malformations.

	No. of study	Summary RR (95%CI)	I^2^ (%)	P*	P**
Overall	18	1.26 (1.13–1.39)	53.6	0.003	
Cardiac malformation	7	1.17 (1.06–1.28)	31.0	0.19	
ASD	6	2.06 (1.40–3.03)	57.8	0.04	
VSD	7	1.15 (0.97–1.36)	30.3	0.20	
ASD and/or VSD	14	1.27 (1.14–1.42)	40.0	0.04	
Geographic Location					0.52
Northern America	5	1.22 (1.12–1.34)	0.0	0.51	
Europe	11	1.27 (1.08–1.48)	67.3	0.001	
Others	2	1.71 (1.23–2.37)	0.0	0.45	
Adjustment for confounders					
Maternal age					0.39
Yes	13	1.23 (1.07–1.41)	62.7	0.001	
No	5	1.34 (1.13–1.59)	18.0	0.30	
Socioeconomic status					0.23
Yes	4	1.39 (0.97–2.00)	75.1	0.007	
No	14	1.19 (1.09–1.30)	29.5	0.14	
Smoking or alcohol drinking					0.72
Yes	9	1.24 (1.05–1.47)	72.7	<0.001	
No	9	1.27 (1.14–1.41)	6.0	0.39	
Pregnancy BMI					0.29
Yes	3	1.10 (0.89–1.36)	0.0	0.70	
No	15	1.29 (1.15–1.46)	62.2	0.001	
Pregnancy complications					0.06
Yes	6	1.13 (1.05–1.22)	0.0	0.96	
No	12	1.40 (1.17–1.67)	63.1	0.002	
Parity					0.73
Yes	6	1.31 (1.05–1.63)	82.0	<0.001	
No	12	1.24 (1.14–1.34)	0.0	0.47	

*P for heterogeneity within each subgroup.

**P for heterogeneity between subgroups with meta-regression analysis.

Abbreviations: ASD, atrial septal defect; BMI, body mass index; CI, confidence interval; RR, relative risk; VSD, ventricular septal defect.
