# Supplementary material for: Use of selective serotonin-reuptake inhibitors in the first trimester and risk of cardiovascular-related malformations: a meta-analysis of cohort studies
Source: Sci Rep. 2017 Feb 21;7:43085. doi: 10.1038/srep43085 (PMC5318893; doi:10.1038/srep43085)
Supplement: Supplementary Tables [file srep43085-s1.pdf]

Use of selective serotonin-reuptake inhibitors in the first trimester and risk of cardiovascular-related malformations: a meta-analysis of cohort studies

Tie-Ning Zhang[1], Shan-Yan Gao[2], Zi-Qi Shen[3], Da Li[3], Cai-Xia Liu[3], Hai-Chen Lv[4], Yuan Zhang[5], Ting-Ting Gong[3], Xin Xu[2], Chao Ji[2], Qi-Jun Wu[2,*]

Supplementary Table S1. Characteristics of cohort studies in the meta-analysis

First author (ref), year, location	No. of cohort/cases	Study period	Outcomes	Risk estimates (95% CI)	Adjusted factors
Furu et al [30], 2015, Europe	2,303,647	1996-2010		Odds Ratio	Maternal age, year of birth, birth order, smoking, maternal diabetes, country, and use of other prescribed drugs.
	27,309		Any cardiac defect	1.15 (1.05-1.26)	
	17,879		ASD and VSD	1.17 (1.05-1.31)	
	1,095		Atrioventricular septal defect	1.22 (0.77-1.91)	
	2,261		Conotruncal and major arch Anomalies	0.95 (0.67-1.35)	
	2,641		Left ventricular outflow tract Obstruction	1.09 (0.80-1.49)	
	2,778		Right ventricular outflow tract Obstruction	1.48 (1.15-1.89)	
	657		Situs anomalies and looping defect	1.00 (0.52-1.90)	
Berard et al [31], 2015, North America	18,493	1998-2010		Risk Ratio	Maternal age, welfare status, diabetes, . hypertension,asthma,and other medication use
	405		Cardiac malformations	1.10 (0.82-1.48)	
	322		ASD/VSD	1.30 (0.99-1.70)	

Supplementary Table S1. Continued

First author (ref), year, location	No. of cohort/cases	Study period	Outcomes	Risk estimates (95% CI)	Adjusted factors
Ban et al [32], 2014, Europe	349,127	1990-2009		Odds Ratio	Maternal age at the end of pregnancy, year of childbirth,townsend deprivation quintile, maternal smoking history,BMI before pregnancy, and maternal diabetes,hypertension, asthma, and epilepsy in the year before conception or during pregnancy.
	2,512		Specific heart anomalies	1.14 (0.89-1.45)	
	N/A		Septal defect	0.89(0.63-1.27)	
	N/A		ASD	1.68 (0.98-2.91)	
	N/A		VSD	0.63(0.38-1.03)	
	N/A		LVOTD	1.05 (0.20-11.24)	
	N/A		RVOTD	2.22 (0.98-5.03)	
Huybrechts et al [33], 2014, North America	949,504	2000-2007		Odds Ratio	N/A
	6,819		Cardiac malformations	1.25 (1.13-1.38)	
	3,413		VSD	1.20 (1.04-1.39)	
	1,106		Right ventricular outflow tract obstruction	1.12 (0.87-1.45)	
	3,458		Other cardiac defect	1.34 (1.17-1.54)	

Supplementary Table S1. Continued

First author (ref), year, location	No. of cohort/cases	Study period	Outcomes	Risk estimates (95% CI)	Adjusted factors
Knudsen et al [34], 2014, Europe	72,280	1995-2008		Odds Ratio	Maternal age, year of conception, use of antiepileptics and/or insulin during first trimester.
	546		CHD	1.64 (0.89-3.00)	
	129		Severe CHD	4.03 (1.75-9.26)	
	97		ASD	2.82 (0.88-9.04)	
	333		VSD	0.94 (0.35-2.53)	
	54		Pulmonary valve stenosis	3.47 (0.83-14.5)	
Vasilakis-Scaramozza et al [35], 2013, Europe	9,893	1991-2002		Relative Risk	Prepregnancy BMI, maternal age, cigarette smoking status, history of DM, insulin use, exposure to a teratogen during the first trimester, history of infertility, and premature delivery.
	35		Cardiovascular anomalies	0.80 (0.40-1.90)	
Margulis et al [36], 2013, Europe	149,464	1996-2010		Odds Ratio	Year of delivery, maternal age at delivery, prepregnancy marital status, index of multiple deprivation at the practice level, family history of congenital malformations, prepregnancy body mass, prepregnancy diabetes, cigarette smoking, alcohol intake, diagnosis of depression in baseline year, diagnosis of other mental conditions in baseline year or contact with or referral to a psychiatrist, number of health care encounters in baseline year and number of nonantidepressant drugs prescribed in baseline year.
	721		Cardiac malformations	1.09 (0.66-1.79)	
	N/A		Septal defects	1.12 (0.60-2.09)	

Supplementary Table S1. Continued

First author (ref), year, location	No. of cohort/cases	Study period	Outcomes	Risk estimates (95% CI)	Adjusted factors
Nordeng et al [38], 2012,	63,395	1999-2009		Odds Ratio	Maternal depression, maternal age at delivery, parity, and use of psychotropic drugs during
Europe	547		Cardiovascular anomalies	1.51 (0.67-3.43)	pregnancy.
	N/A		ASD/VSD	1.57 (0.64-3.87)	
Jimenez-	848,786	1997-2009		Odds Ratio	Mother's age, parity, income, education, smoking,
Solem et al [39], 2012, Europe	7,832		Congenital malformation of heart	2.01 (1.60-2.53)	And year of conception.
	2,524		ASD	2.60 (1.84-3.68)	
	N/A		Atrioventricular sepetal defect	1.25 (0.31-5.02)	
	2,824		VSD	1.62 (1.05-2.50)	
	4,875		Septal defect	2.04 (1.53-2.72)	
Colvin et al [21],	123,405	2002-2005		Odds Ratio	N/A
2011, other regions	691		Cardiovascular anomalies	1.60 (1.10-2.31)	
	97		Ostium secundum type ASD	2.73 (1.26-5.89)	
	218		VSD	0.99 (0.44-2.23)	

Supplementary Table S1. Continued

First author (ref), year, location	No. of cohort/cases	Study period	Outcomes	Risk estimates (95% CI)	Adjusted factors
	382		Bulbus cordis anomalies and anomalies of cardiac septal closure	1.23 (0.71-2.15)	
	250		Other congenital anomoly of heart	1.77 (0.99-3.16)	
Malm et al [37], 2011, Europe	635,583	1996-2006		Odds Ratio	Age at the end of pregnancy, parity, year of pregnancy ending, smoking during pregnancy, purchase of other reimbursed psychiatric drugs during the first trimester, and maternal prepregnancy diabetes.
	8,253		All major cardiovascular anomalis	1.09 (0.90-1.32)	
	1,297		ASD	1.04 (0.64-1.69)	
	5,550		VSD	1.20 (0.96-1.50)	
	437		Conotruncal heart defect	0.46 (0.14-1.46)	
	476		Left ventricular outflow tract defects	0.93 (0.40-2.13)	
	423		Right ventricular outflow tract defects	1.74 (0.85-3.57)	
	240		Transposition of great arteries	0.60 (0.15-2.52)	

Supplementary Table S1. Continued

First author (ref), year, location	No. of cohort/cases	Study period	Outcomes	Risk estimates (95% CI)	Adjusted factors
Kornum et al [40], 2010, Europe	216,042	1991-2007		Odds Ratio	Maternal smoking status, maternal age, birth order, and birth year.
	1,429		Cardiac malformations	1.70 (1.10-2.50)	
	N/A		Septal heart defect	1.40 (0.80-2.30)	
Merlob et al [20],2009, Other regions	67,871	2000-2007		Relative Risk	N/A
	1,091		Congenital heart malformation	2.17 (1.07-4.39)	
Pedersen et al [19], 2009, Europe	493,113	1996-2003		Odds Ratio	Age, calendar year, income, marriage status, tobacco smoking.
	4,004		Major cardiac malformations	1.44 (0.86-2.40)	
	2,327		Septal heart defects	1.99 (1.13-3.53)	
Oberlander et al [22], 2008, North America	11,957	1998-2001		Odds Ratio	N/A
	529		Cardiovascular congenital defects	1.36 (0.84-2.21)	
	81		ASD	3.28 (1.42-7.53)	
	225		VSD	1.12 (0.50-2.52)	

Supplementary Table S1. Continued

First author (ref), year, location	No. of cohort/cases	Study period	Outcomes	Risk estimates (95% CI)	Adjusted factors
Davis et al [41], 2007, North America	87,407	1996-2000		Relative Risk	Health system, maternal age, and birth season.
	682		Bulbus cordis anomalies and anomalies of cardiac septal closure	0.93 (0.50-1.73)	
	506		Other congenital anomolies of heart	0.88 (0.42-1.86)	
Kallen et al [42], 2007, Europe	880,431	1995-2004		Odds Ratio	Year of birth, maternal age, parity, smoking, and≥3 Previous miscarriages.
	11,445		Any cardiac defect	0.97 (0.77-1.21)	
	7,213		VSD and/or ASD	1.10 (0.84-1.44)	
	1,218		Unspecified cardiac defect	1.04 (0.42-2.15)	
Kulin et al [43], 1998, North America	534			Relative Risk	N/A
	6		Cardiac malformations	0.53 (0.10-2.86)	

Abbreviations: ASD, atrial septal defect; BMI, body mass index; CHD, congenital heart defect; DM, diabetes mellitus; LVOTD, left ventricular outflow tract defect; N/A, not available; RVOTD, right ventricular outflow tract defect; VSD, ventricular septal defect.

Supplementary Table S2. Methodological quality of cohort studies included in the meta-analysis[a]

First author (reference), publication year	Representativeness of the exposed cohort	Selection of the unexposed cohort	Ascertainment of exposure	Outcome of interest not present at start of study	Control for important factor or additional factor [b]	Assessment of outcome	Follow-up long enough for outcomes to occur[c]	Adequacy of follow-up of cohorts [d]
Furu et al [30], 2015	*	*	*	*	**	*	*	*
Berard et al [31], 2015	*	*	*	*	*	*	*	*
Ban et al [32], 2014	*	*	*	*	**	*	-	-
Huybrechts et al [33], 2014	*	*	*	*	-	*	*	*
Knudsen et al [34], 2014	*	*	*	*	*	*	*	*
Vasilakis-Scaramozza et al [35], 2013	*	*	*	*	**	*	*	*
Margulis et al [36], 2013	*	*	*	*	**	*	*	*
Nordeng et al [38], 2012	*	*	*	*	*	*	-	-
Jimenez-Sole et al [39], 2012	*	*	*	*	**	*	*	*
Colvin et al [21], 2011	*	*	*	*	-	*	*	*
Malm et al [37], 2011	*	*	*	*	**	*	-	-
Kornum et al [40], 2010	*	*	*	*	**	*	*	*
Merlob et al [20], 2009	*	*	*	*	-	*	*	*
Pedersen et al [19], 2009	*	*	*	*	**	*	*	*
Oberlander et al [22], 2008	*	*	*	*	-	*	-	-
Davis et al [41], 2007	*	*	*	*	*	*	*	*
Kallen et al [42], 2007	*	*	*	*	**	*	-	-
Kulin et al [43], 1998	*	*	*	*	-	*	-	-

[a] A study could be awarded a maximum of one star for each item except for the item Control for important factor or additional factor. The definition/explanation of each column of the Newcastle-Ottawa Scale is available from (http://www.ohri.ca/programs/clinical_epidemiology/oxford.asp.).

[b] A maximum of 2 stars could be awarded for this item. Studies that controlled for age received one star, whereas studies that controlled for other important confounders such as smoking and/or alcohol using received an additional star.

[c] A cohort study with a follow-up time >9 months was assigned one star.

[d] A cohort study with a follow-up rate >75% was assigned one star.
